# The E3 ligase OsPUB15 interacts with the receptor-like kinase PID2 and regulates plant cell death and innate immunity

**DOI:** 10.1186/s12870-015-0442-4

**Published:** 2015-02-13

**Authors:** Jing Wang, Baoyuan Qu, Shijuan Dou, Liyun Li, Dedong Yin, Zhiqian Pang, Zhuangzhi Zhou, Miaomiao Tian, Guozhen Liu, Qi Xie, Dingzhong Tang, Xuewei Chen, Lihuang Zhu

**Affiliations:** State Key Laboratory of Plant Genomics and National Center for Plant Gene Research, Institute of Genetics and Developmental Biology, Chinese Academy of Sciences, Beijing, 100101 China; Rice Research Institute, Sichuan Agricultural University, Chengdu, Sichuan 611130 China; State Key Laboratory for Plant Cell and Chromosome Engineering, Institute of Genetics and Developmental Biology, Chinese Academy of Sciences, Beijing, 100101 China; College of Life Sciences, Hebei Agricultural University, Baoding, Hebei 071001 China; CAS Key Laboratory of Genome Sciences and Information, Beijing Institute of Genomics, Chinese Academy of Sciences, Beijing, 100029 China

**Keywords:** U-box, E3 ligase, Protein interaction, Blast resistance, Cell death, Rice

## Abstract

**Background:**

Rice blast disease is one of the most destructive diseases of rice worldwide. We previously cloned the rice blast resistance gene *Pid2*, which encodes a transmembrane receptor-like kinase containing an extracellular B-lectin domain and an intracellular serine/threonine kinase domain. However, little is known about *Pid2*-mediated signaling.

**Results:**

Here we report the functional characterization of the U-box/ARM repeat protein OsPUB15 as one of the PID2-binding proteins. We found that OsPUB15 physically interacted with the kinase domain of PID2 (PID2K) *in vitro* and *in vivo* and the ARM repeat domain of OsPUB15 was essential for the interaction. *In vitro* biochemical assays indicated that PID2K possessed kinase activity and was able to phosphorylate OsPUB15. We also found that the phosphorylated form of OsPUB15 possessed E3 ligase activity. Expression pattern analyses revealed that *OsPUB15* was constitutively expressed and its encoded protein OsPUB15 was localized in cytosol. Transgenic rice plants over-expressing *OsPUB15* at early stage displayed cell death lesions spontaneously in association with a constitutive activation of plant basal defense responses, including excessive accumulation of hydrogen peroxide, up-regulated expression of pathogenesis-related genes and enhanced resistance to blast strains. We also observed that, along with plant growth, the cell death lesions kept spreading over the whole seedlings quickly resulting in a seedling lethal phenotype.

**Conclusions:**

These results reveal that the E3 ligase OsPUB15 interacts directly with the receptor-like kinase PID2 and regulates plant cell death and blast disease resistance.

**Electronic supplementary material:**

The online version of this article (doi:10.1186/s12870-015-0442-4) contains supplementary material, which is available to authorized users.

## Background

Plants respond to pathogen infection using their innate immunity system, which includes two layers, pathogen/microbe-associated molecular pattern (PAMP/MAMP)-triggered immunity (PTI) and effector-triggered immunity (ETI) [1,2]. PTI, also known as the plant basal defense, is mediated by plant pattern recognition receptors (PRRs) through recognizing the conserved microbial features PAMPs/MAMPs [3,4]. Compared to ETI, PTI mediates a relatively weaker immune response with broad-spectrum defense against pathogens. There are some typical downstream responses in this pathway, including the activation of mitogen-activated protein kinases (MAPKs), rapid production of reactive oxygen species (ROS) and the induction of pathogenesis-related (PR) genes [5-7]. When pathogens develop specific effectors to suppress PTI, plants evolve corresponding resistance proteins to recognize these effectors and lead to ETI, a much more rapid and robust immune response [8]. Besides the signaling process present in PTI, hypersensitive response (HR), a form of localized programmed cell death (PCD) at the infection sites of plant to prevent the spread of pathogens, is usually accompanied with ETI [9]. Some plant mutants develop spontaneous cell death lesions similar to HR cell death in the absence of pathogen infection and such mutants are called lesion mimic mutants [10,11]. There are a lot of overlaps in the signal pathways of lesion mimic PCD and plant innate immunity, suggesting the existence of signaling crosstalk between them [12,13].

Protein degradation mediated by the ubiquitin-proteasome system (UPS) plays critical roles in plant immunity. UPS is used for selectively degrading proteins through a process of polyubiquitination in eukaryotic organisms. Ubiquitination of a target protein is carried out by attaching ubiquitin molecules to the target through a sequential action of ubiquitin-activating enzyme (E1), ubiquitin-conjugating enzyme (E2) and ubiquitin protein ligase (E3). The protein conjugated with a poly-ubiquitin chain is then targeted to the 26S proteasome for degradation [14,15]. Among the UPS components, E3 ligases are the most diverse members as they determine the substrate specificity [16,17]. E3 ligases can be classified into different groups based on the presence of specific HECT, RING, or U-box domains [18,19]. The U-box domain is a modified RING domain consisting of ~70 conserved amino acids [20,21]. In both rice (*Oryza sativa* L.) and *Arabidopsis thaliana*, the largest group of predicted plant U-box proteins (PUBs) contains varying repeats of C-terminal armadillo (ARM) motif which are involved in the protein-protein interactions [22-24]. Many plant U-box/ARM repeat proteins have been identified as active E3 ligases with important roles in diverse biological processes, such as self-incompatibility [25,26], plant hormone responses [27-30], abiotic stresses [31-35], flowering time [36,37], plant cell death and defense responses [36,38-42].

Rice blast disease, caused by the fungus of *Magnaporthe oryzae* (*M. oryzae*), is one of the most destructive diseases of rice worldwide [43]. To date, more than two dozens of rice blast resistance genes have been cloned and characterized, but the immediate downstream signaling events mediated by these resistance genes are largely unknown. The rice blast resistance gene *Pid2* encodes a transmembrane B-lectin receptor-like kinase [44]. To identify the interaction partners of PID2, we formerly used the intracellular kinase domain of PID2 as bait to screen a rice cDNA library via yeast two-hybrid approach and obtained some binding proteins [45]. Among them, a protein encoded by *LOC*_*Os08g01900* was previously reported as a U-box/ARM repeat protein called OsPUB15 [23].

Here, we report that OsPUB15 is able to interact directly with the kinase domain of PID2 *in vitro* and *in vivo*. Our study also reveals that OsPUB15 could be phosphorylated by PID2K in an ARM-dependent manner and the phosphorylated OsPUB15 exhibits E3 ligase activity. Furthermore, overexpression of *OsPUB15* in rice leads to a spontaneous cell death phenotype and a constitutive activation of plant basal defense response. These findings demonstrate that OsPUB15 plays critical roles in plant cell death and innate immunity.

## Results

### OsPUB15 is a U-Box/ARM repeat protein

**Figure 1:**
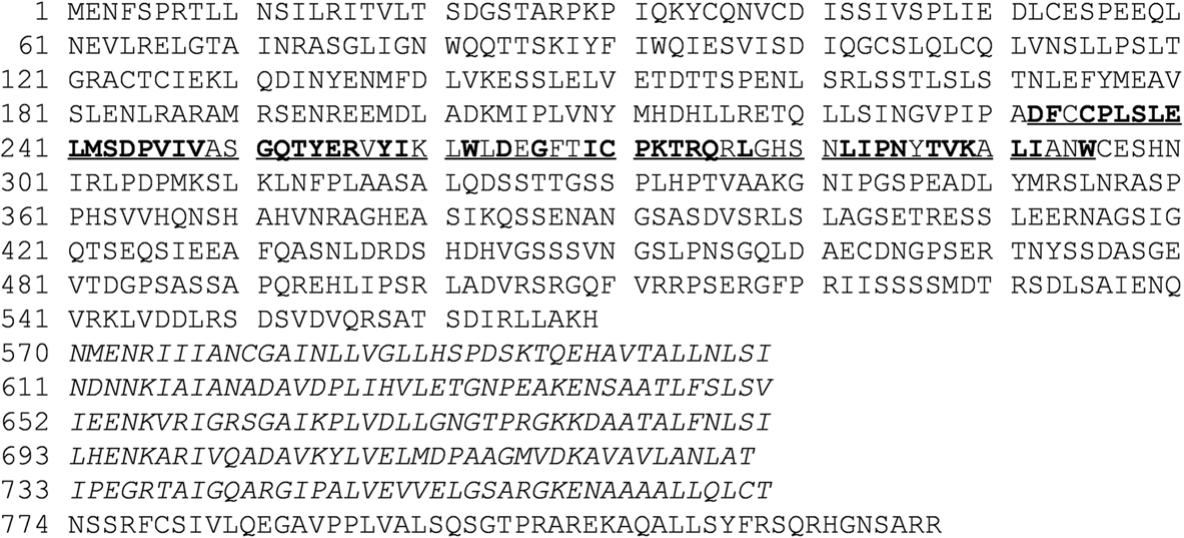
**Predicted amino acid** **(aa)** **sequence of OsPUB15.** The U-box domain from amino acid 232 to 295 is underlined, in which the conserved amino acids are indicated in bold. Five ARM repeat motifs in the C-terminal region are separately shown in italics.

The previous study identified OsPUB15 as one of the PID2K-interacting proteins through yeast two-hybrid screening [45]. According to the rice genome annotation database (http://rice.plantbiology.msu.edu), OsPUB15 is annotated as a putative ARM (armadillo) repeat family protein composed of 824 amino acids with a molecular mass of approximate 90 kD. The SMART (http://smart.embl-heidelberg.de/) database shows that OsPUB15 contains a conserved U-box domain spanning the amino acid residues 232 to 295 and five tandem repeats of ARM motifs in its C terminus (Figure 1). Phylogenetic analysis on the ARM repeat-containing PUBs revealed that OsPUB15 was most closely related to NtPUB4 (51.8% sequence identity) and AtPUB4 (50.4% sequence identity, Additional file 1: Figure S1). NtPUB4 was reported to interact directly with the receptor-like kinase CHRK1 and it was predicted to be involved in modulating the plant developmental signaling pathway mediated by CHRK1 [46]. AtPUB4 was identified to influence male fertility through impacting growth and degeneration of tapetal cells of *Arabidopsis* [47]. In rice, OsPUB15 was grouped into cluster I of the rice U-box/ARM repeat protein subfamily [22,23] and it was found to share the highest sequence similarity (50.2% identity, 65.7% similarity) with OsPUB16, an uncharacterized PID2K-interacting protein [45].

### The ARM repeat domain of OsPUB15 is required for directly interacting with PID2K

**Figure 2:**
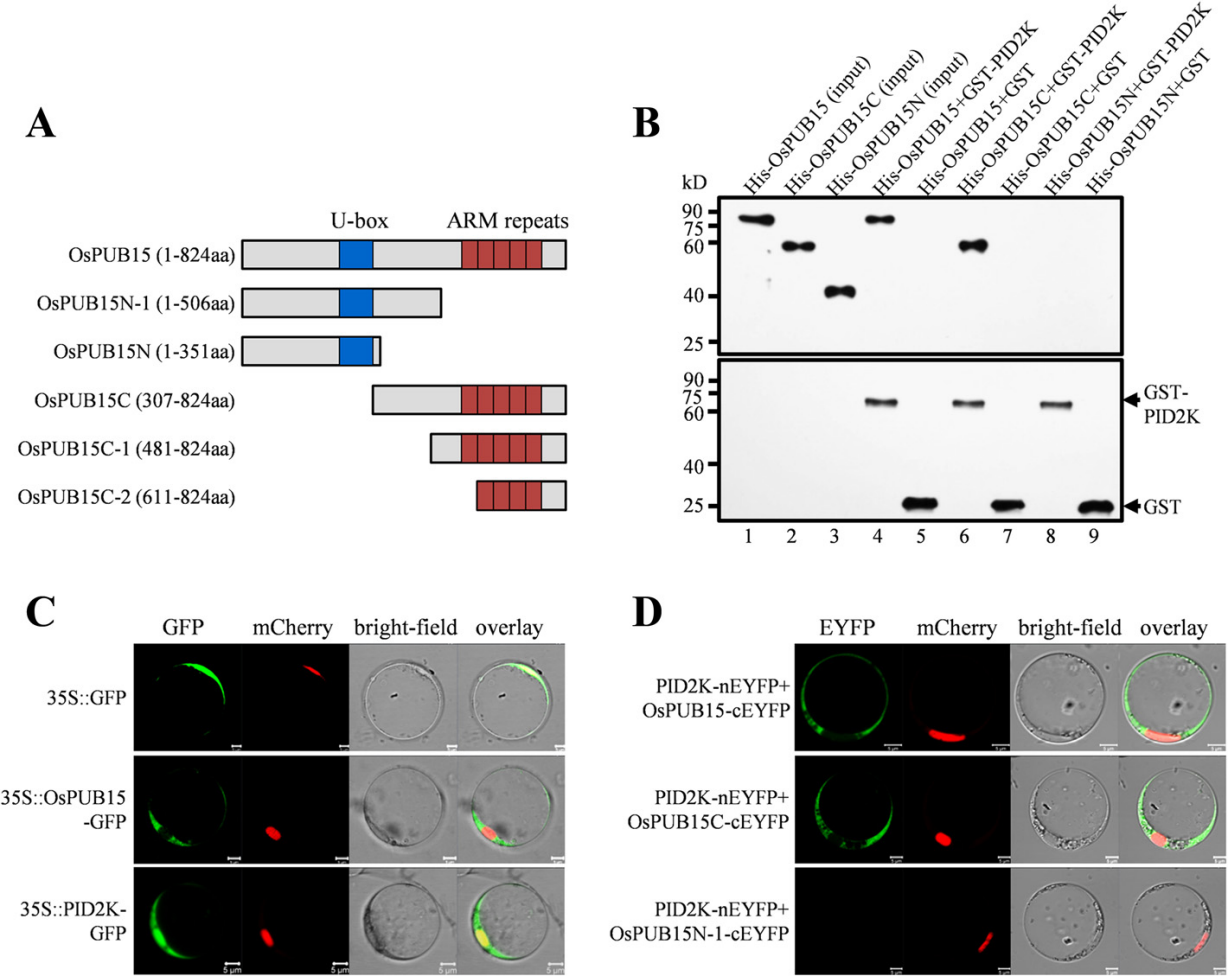
**Determination of interactions between OsPUB15 variants and PID2K. (A)** Schematic representations of OsPUB15 and its truncated variants. **(B)** *In vitro* binding analysis of OsPUB15 and its truncated variants to the PID2 kinase domain. The purified His-fusion proteins were separately mixed with equal quantities of resin bound GST-PID2K and then pulled down by the resin. After extensive washings, the pull-down products were immunoblotted with anti-OsPUB15 (top panel) or anti-GST (bottom panel) antibody. One-tenth of the input of purified His fusions, His-OsPUB15 (lane 1), His-OsPUB15C (lane 2) and His-OsPUB15N (lane 3), were loaded as controls. **(C)** Subcellular localization of OsPUB15 and PID2K, respectively. Rice protoplasts were co-transformed with the nuclear marker mCherry-VirD2NLS (mCherry-NLS) accompanied with GFP alone (top panel) or GFP fused proteins (middle and bottom panel) driven by 35S promoter, respectively. The panels from left to right are confocal micrographs of GFP signal (green), mCherry signal (red), bright-field images and the resultant overlaid images, respectively. Bars = 5 μm. **(D)** BiFC analysis of *in vivo* interaction between PID2K and OsPUB15 or its variants. PID2K and the OsPUB15 variants were respectively fused to the inactive N-terminal (nEYFP) or the C-terminal (cEYFP) part of EYFP, and the pairs of indicated recombinant proteins were transiently expressed in rice protoplasts along with mCherry-VirD2NLS (mCherry-NLS), a nuclear marker. The fluorescence signals were monitored by confocal microscopy. The panel shows fluorescence images of the EYFP signal (green), the mCherry signal (red), the bright-field illumination of protoplasts and the overlaid images, respectively. Bars = 5 μm. The above experiments were repeated three times with similar results.

To confirm the interaction between OsPUB15 and PID2K detected from the yeast-two-hybrid system, we performed *in vitro* pull down assays. For these assays, PID2K was expressed in frame with Glutathione S transferase (GST) in yeast and OsPUB15 was expressed as a His-tagged fusion protein in *Escherichia coli* (*E. coli*), respectively. The expressed GST-PID2K was then purified with Glutathione Sepharose 4B beads and incubated with the purified His-OsPUB15. Following extensive washings, the proteins bound to the beads were separated by SDS-PAGE and immunoblotted with anti-OsPUB15 and anti-GST antibodies, respectively. We found that the anti-OsPUB15 antibody was able to specifically detect a clear band about 90 kD as the size of OsPUB15 from the components pulled down by GST-PID2K (Figure 2B, lane 4) whereas no band was detected from those pulled down by GST alone (Figure 2B, lane 5). This result reveals that OsPUB15 is able to interact with PID2K directly, which is consistent with the result obtained from yeast-two-hybrid.

In order to determine the fragment of OsPUB15 which is essential for its interaction with PID2K, two OsPUB15 variants, OsPUB15C (C-terminus of OsPUB15, amino acids 307-824) and OsPUB15N (N-terminus of OsPUB15, amino acids 1-351, Figure 2A) were separately expressed as His-fused proteins and the purified His-OsPUB15C and His-OsPUB15N were respectively introduced into GST pull-down assays. We found that the anti-OsPUB15 antibody was able to detect a positive band from the pulled down components of GST-PID2K post incubation with His-OsPUB15C (Figure 2B, lane 6) rather than with His-OsPUB15N (Figure 2B, lane 8), whereas no band was detected from the pulled down components of GST post incubation with His-OsPUB15C or His-OsPUB15N (Figure 2B, lane 7 and lane 9). These results suggest that the C-terminal fragment containing the entire ARM repeat domain of OsPUB15 is essential and sufficient for the interaction between OsPUB15 and PID2K.

To further verify the interaction between OsPUB15 and PID2K, we performed bimolecular fluorescence complementation (BiFC) assays. Before this analysis, we firstly determined the subcellular localization of OsPUB15 and PID2K, individually. We fused the coding region of OsPUB15 and PID2K to the N-terminus of green fluorescent protein (GFP), respectively, to produce the OsPUB15-GFP and PID2K-GFP fusion proteins. Each of these fusion proteins under the control of CaMV 35S promoter was co-expressed with the nuclear marker mCherry-VirD2NLS in rice protoplasts, respectively. We found that the fluorescence signals were present at cytosol when the protoplast cells were transformed with OsPUB15-GFP, whereas the fluorescence signals were present at cytosol and nucleus when the protoplast cells were transformed with PID2K-GFP (Figure 2C). These results suggest that OsPUB15 is located in cytosol while PID2K, the separated intracellular part of PID2, distributes in both the nucleus and the cytosol of rice cells.

To further determine the domain required for OsPUB15 to interact with PID2K *in vivo*, we created another variant called OsPUB15N-1 (amino acids 1-506, Figure 2A), which is longer than OsPUB15N, and applied it to the following BiFC assays. PID2K and the variants of OsPUB15 were respectively fused to the split N-terminal (nEYFP) and C-terminal (cEYFP) fragments of enhanced yellow fluorescent protein (EYFP) to generate the constructs, PID2K-nEYFP, OsPUB15-cEYFP, OsPUB15C-cEYFP and OsPUB15N-1-cEYFP. The pairs, PID2K-nEYFP/OsPUB15-cEYFP, PID2K-nEYFP/OsPUB15C-cEYFP and PID2K-nEYFP/OsPUB15N-1-cEYFP, were respectively co-transformed with mCherry-VirD2NLS into rice protoplasts. We detected the fluorescence signals in cytosol when the pair of PID2K-nEYFP/OsPUB15-cEYFP was co-transformed into protoplasts (Figure 2D), suggesting that OsPUB15 did interact with PID2K. This result is consistent with the cytosolic co-localization of both PID2K and OsPUB15 (Figure 2C). Moreover, the fluorescence signals were also appeared in the cytosol of the protoplasts transformed with PID2K-nEYFP/OsPUB15C-cEYFP, but not in the protoplasts transformed with PID2K-nEYFP/OsPUB15N-1-cEYFP (Figure 2D), suggesting that the variant, OsPUB15C but not OsPUB15N-1 interacts with PID2K. Together with the sequences and structures of OsPUB15C and OsPUB15N-1 (Figure 2A), these results also suggest that the ARM domain of OsPUB15 is essential for the interaction of OsPUB15 with PID2K *in vivo*.

### OsPUB15 is transphosphorylated by PID2K in an ARM-dependent manner

**Figure 3:**
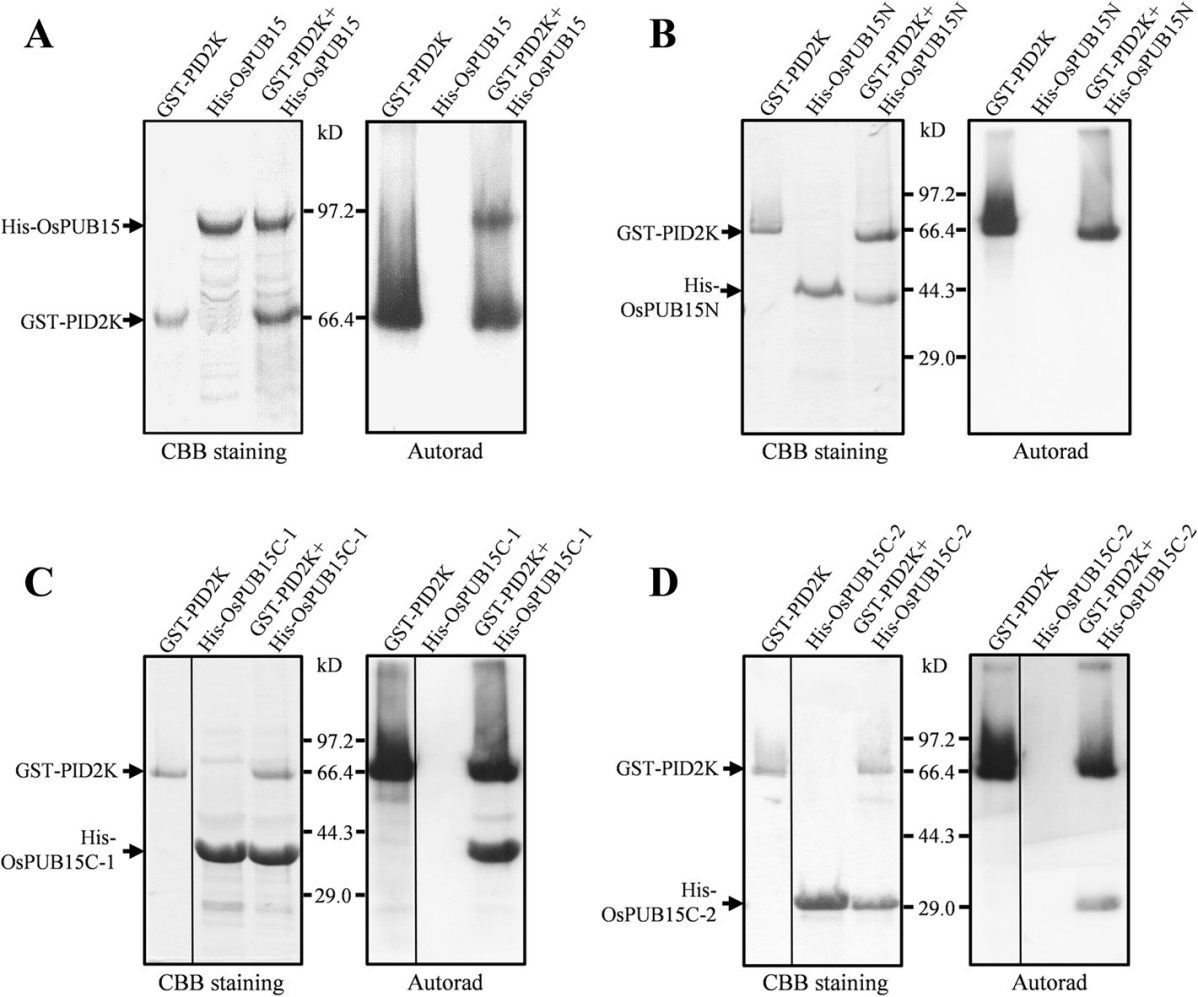
**The ARM repeat domain is required for OsPUB15 to be phosphorylated by PID2K.** *In vitro* phosphorylation analysis of OsPUB15 **(A)**, OsPUB15N **(B)**, OsPUB15C-1 **(C)** or OsPUB15C-2 **(D)** by the kinase domain of PID2. The purified His fusion proteins were incubated with purified GST-PID2K in the presence of [γ-^32^P] ATP. GST-PID2K was used as a positive control. Samples were separated with SDS-PAGE followed by Coommassie Brilliant Blue staining (CBB staining) and Autoradiography (Autorad), respectively. Similar results were obtained from three independent experiments.

Since PID2K is the putative kinase domain of PID2 [44], we were interested in determining whether PID2K really possessed kinase activity. For this purpose, we performed *in vitro* phosphorylation assay on the purified protein GST-PID2K and found that the PID2K was capable of autophosphorylation (Figure 3A). This result suggests that PID2 is an active kinase. As OsPUB15 could interact with PID2K directly, we wondered whether PID2K was capable of phosphorylating OsPUB15. We then performed a similar kinase activity analysis on GST-PID2K co-incubated with the purified His-OsPUB15. We found that not only GST-PID2K but also His-OsPUB15 was phosphorylated, whereas, His-OsPUB15 was not phosphorylated when it was incubated in the absence of GST-PID2K (Figure 3A). Our results indicate that PID2K is able to transphosphorylate OsPUB15 and thus OsPUB15 is a substrate of the active kinase PID2K.

To examine whether the phosphorylation sites of OsPUB15 are in the ARM repeat domain as it is responsible for interacting with PID2K, we conducted additional phosphorylation assays using the OsPUB15 variants as substrates. To avoid the potential confusion likely caused by the same molecular weight (65 kD) of His-OsPUB15C and GST-PID2K, His-OsPUB15C-1 (amino acids 481-824, 45 kD), with different molecular weight, was created and used in the assays. We found that His-OsPUB15C-1 could be efficiently phosphorylated by GST-PID2K (Figure 3C), whereas His-OsPUB15N was not (Figure 3B). Furthermore, we also found that the variant OsPUB15C-2 (amino acids 611-824) containing only four ARM motifs was sufficient for OsPUB15 to be phosphorylated by PID2K (Figure 3D). These results suggest that the PID2K-phosphorylated residues are mainly distributed in the ARM repeat domain of OsPUB15.

### Phosphorylation is required for OsPUB15 to exhibit its E3 ligase activity

As U-box proteins usually possess E3 ubiquitin ligase activity [22,28,48,49], we presumed that OsPUB15 might function as an E3 ligase. To test this hypothesis, we performed *in vitro* self-ubiquitination assay on bacterially expressed His-OsPUB15. However, no E3 ligase activity was detected for the purified His-OsPUB15 in the presence of wheat E1, human E2 (UBCh5b) and His-tagged ubiquitin as well as in the reactions lacking of E1, E2 or His-OsPUB15, while the positive control SDIR1 showed strong self-ubiquitination activity evidenced by detection of high-molecular-weight bands with an antibody to ubiquitin (Additional file 1: Figure S2). We then performed additional ubiqutination assays on the purified His-OsPUB15 using ten different *Arabidopsis* E2s, ATS1, ATS2, ATS5, ATS6, ATS8, ATS9, ATS10, ATS12, UBC4 and UBC32, respectively, and found that His-OsPUB15 did not show any detectable E3 ligase activity when incubated with any of these E2s (data not shown).

**Figure 4:**
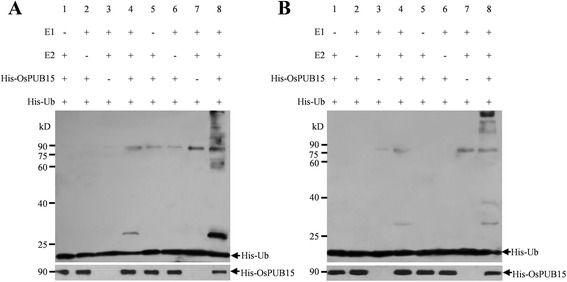
**The pre**-**treated OsPUB15 possesses E3 ligase activity. (A)** E3 ligase activity analysis of pre-incubated His-OsPUB15. The purified His-OsPUB15 was incubated with total protein extracts of rice for one hour, and then was put into self-ubiquitination assays in the presence of wheat E1, human E2 (UBCh5b) and 6xHis tag ubiquitin (His-Ub) or in the absence of E1, E2 or His-OsPUB15 (lanes 5-8). As a control, the un-pre-incubated His-OsPUB15 was included in the assays (lanes 1-4). After the reactions, samples were separated by 12% SDS-PAGE and immunoblotted with anti-Ub (top panel) or anti-OsPUB15 (bottom panel) antibody. **(B)** E3 ligase activity analysis of PID2K-phosphorylated His-OsPUB15. The untreated His-OsPUB15 (lanes 1-4) and the PID2K-phosphorylated His-OsPUB15 (lanes 5-8) were assayed for ubiquitination activity, respectively. The reaction products were immunoblotted with anti-Ub (top panel) or anti-OsPUB15 (bottom panel) antibody. These experiments were repeated three times with similar results obtained.

Considering that post translational modification might be required for the E3 ligase activity of OsPUB15, we pre-incubated the purified His-OsPUB15 with the total protein extracts of rice and used such pre-incubated His-OsPUB15 for the *in vitro* ubiquitination assay. The result showed that such pre-treated His-OsPUB15 displayed E3 ligase activity in the presence of E1, E2, and ubiquitin (Figure 4A, lane 8). By contrast, no protein ubiquitination was detected on untreated His-OsPUB15 (Figure 4A, lane 4) as well as in the reactions lacking of E1, E2, or His-OsPUB15 (Figure 4A, lanes 1-3, 5-7). These findings suggest that post translational modification is essential for OsPUB15 to exhibit its E3 ligase activity.

As OsPUB15 could be phosphorylated by PID2K, it was attractive for us to figure out whether the phosphorylation modification was required for OsPUB15 to be an active E3 ligase. For this purpose, we carried out self-ubiquitination assay on the His-OsPUB15 pre-phosphorylated by GST-PID2K. As expected, the results showed that the phosphorylated form of His-OsPUB15 indeed exhibited ubiquitination activity in the presence of E1, E2, and ubiquitin (Figure 4B, lane 8), whereas no ubiquitination activity was detected on unphosphorylated His-OsPUB15 or in the reactions lacking of E1, E2, or His-OsPUB15 (Figure 4B, lanes 1-7). These results suggest that the phosphorylation modification by PID2K is required for OsPUB15 to release its E3 ligase activity.

### *OsPUB15* is constitutively expressed in rice plants

**Figure 5:**
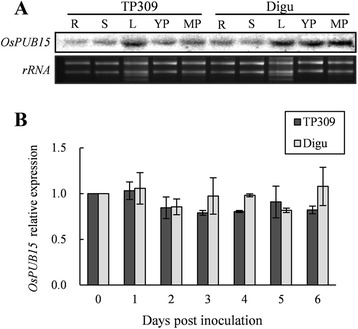
**Expression pattern of** ***OsPUB15*** **. (A)** Northern analysis of *OsPUB15* in different tissues of rice cultivars TP309 and Digu. The total RNAs extracted from various rice tissues were hybridized with ^32^P-labeled *OsPUB15* probe, rRNA signals were used as a sample loading control. R, S, L, YP and MP denote root, stem, leaf, young panicle and mature panicle, respectively. **(B)** qRT-PCR analysis of *OsPUB15* expression profiling in TP309 and Digu at 0, 1, 2, 3, 4, 5 and 6 days post inoculation (DPI) with the blast strain ZB15. The expression level of *OsPUB15* at 0 day was set as 1.0. The expression level of rice *ACTIN1* gene was used as an internal control for normalization of the data. Values are means ± SDs of three biological repeats.

To reveal the expression profile of *OsPUB15*, we performed northern analysis on the RNA samples prepared from diverse tissues, including root, stem, leaf, young panicle and mature panicle of the two rice cultivars, Taipei309 (TP309) and Digu, using the cDNA of *OsPUB15* as the probe. The result shows that *OsPUB15* is ubiquitously expressed in the tested tissues of both rice cultivars, with relatively higher expression level in the leaf of TP309 and in the leaf, young panicle and mature panicle of Digu as well (Figure 5A).

Because PID2 confers resistance to *M. oryzae* isolate ZB15 [44] and OsPUB15 physically interacts with PID2K, we wondered whether the expression of *OsPUB15* was affected by inoculation with ZB15. To address this question, we carried out quantitative real-time PCR (qRT-PCR) analysis to examine the expression level of *OsPUB15* in the resistant rice cultivar Digu and the susceptible cultivar TP309, respectively, post inoculation with ZB15. We found no obvious changes in the expression level of *OsPUB15* in both rice cultivars, which were well but differently responsive to the inoculation as expected (Figure 5B, Additional file 1: Figure S3). This is consistent with the previous result that the expression level of *Pid2* was not affected by inoculation with ZB15 either in susceptible or resistant rice varieties [44].

### Overexpression of *OsPUB15* leads to plant cell death accompanied with excessive accumulation of ROS

**Figure 6:**
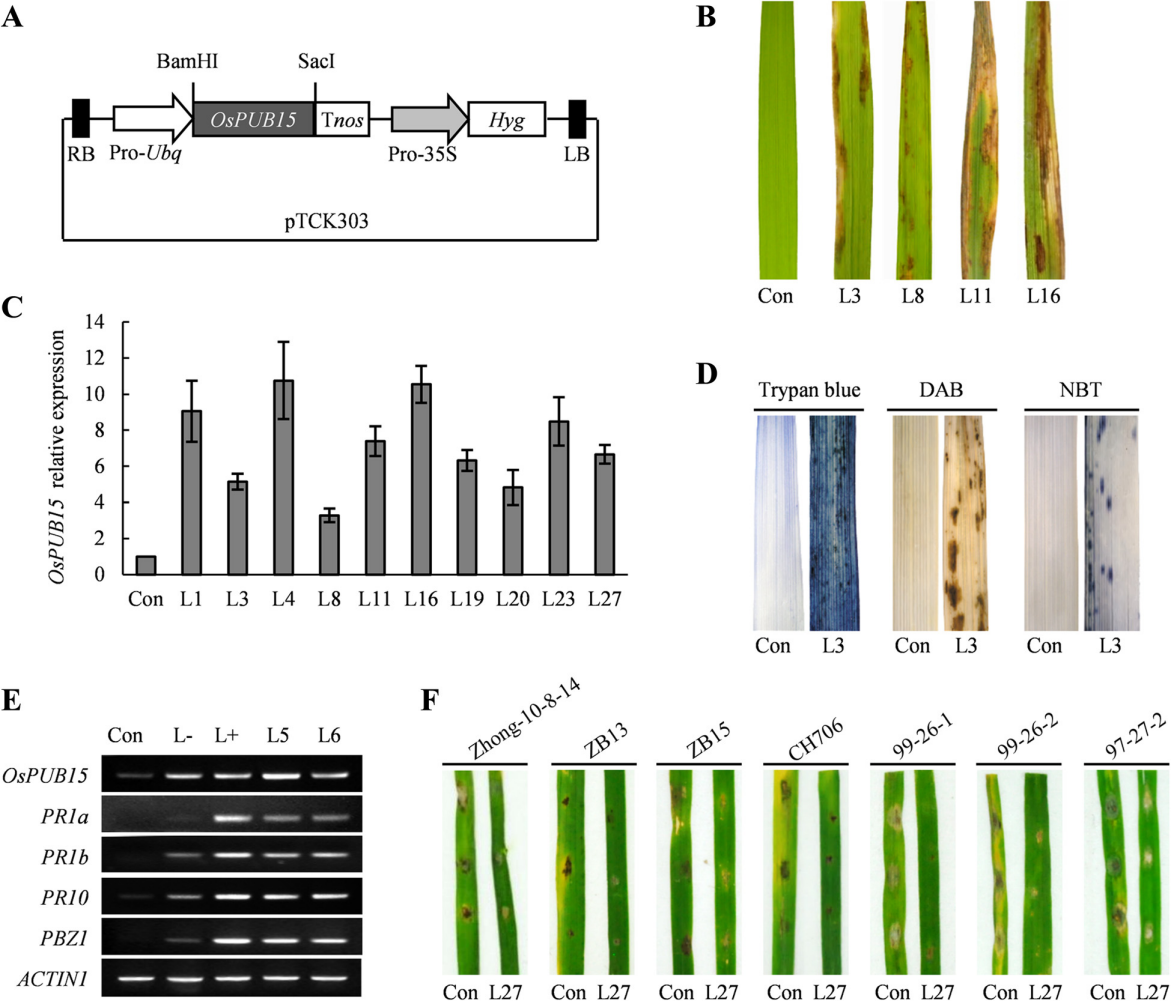
**Phenotypic characterization of the transgenic rice plants over**-**expressing** ***OsPUB15*** **. (A)** Schematic diagram of the *OsPUB15* overexpression vector. The ORF fragment of *OsPUB15* is placed between the maize *Ubiquitin* promoter (Pro-*Ubq*) and *nos* terminator (T*nos*). The *Hyg* gene driven by the CaMV 35S promoter (Pro-35S) is included for hygromycin resistance. **(B)** Spontaneous cell death phenotype of transgenic rice plants over-expressing *OsPUB15* (referred to simply as *OsPUB15*ox) under sterile conditions. L3, L8, L11 and L16 indicate the corresponding *OsPUB15*ox lines while Con represents the control plants expressing the empty vectors. **(C)** qRT-PCR analysis of *OsPUB15* expression in two-week-old *OsPUB15*ox plants and the control plants. The expression level of *OsPUB15* in the control plants was set as 1.0. The expression level of the rice *ACTIN1* gene was used as an internal control for normalization of the data. Data represent means ± SDs of three replicates. **(D)** Histochemical assays of the *OsPUB15*ox plants and the control plants. Leaves of two-week-old transgenic plants were stained with trypan blue (left panel), DAB (middle panel) or NBT (right panel) to indicate cell death, superoxide or hydrogen peroxide accumulation, respectively. L3 and Con represent *OsPUB15*ox line 3 and the control plants, respectively. **(E)** Transcript expression analysis of *PR* genes in transgenic rice plants. Total RNA was extracted from leaves of two-week-old transgenic plants. RT-PCR was performed with specific primers for *PR1a*, *PR1b*, *PR10* and *PBZ1*, respectively. Control RT-PCR reactions were conducted with rice *ACTIN1*. Con, L-, L+, L5 and L6 represent leaves of the control plants, later-emerged young leaves of *OsPUB15*ox before lesion formation, early-emerged old leaves of *OsPUB15*ox with lesions, total leaves of *OsPUB15*ox-L5 and *OsPUB15*ox-L6, respectively. **(F)** Disease resistance determination of *OsPUB15*ox seedlings to *M. oryzae* isolates. The control rice seedlings and the *OsPUB15*ox seedlings were treated with indicated blast strains, and the responses were analyzed two days later. The above experiments were repeated three times with similar results obtained.

To investigate the biological function of *OsPUB15* in rice, we subcloned the coding region of *OsPUB15* into the binary vector pTCK303 under the control of maize *Ubiquitin* promoter to generate the *OsPUB15* overexpression construct (Figure 6A). We then introduced the construct and the empty vector into the calli derived from TP309, respectively, by *Agrobacterium*-mediated transformation. We obtained more than 30 independent transgenic plants over-expressing *OsPUB15* (hereafter referred to as *OsPUB15*ox) in total. A few days after the regenerated transgenic rice plants produced, some brown lesions appeared spontaneously on the early-emerged old leaves of all the *OsPUB15*ox plants and the lesions spread over the whole seedlings quickly resulting in plants death within one month even under sterile conditions. By contrast, no morphological abnormalities were observed on the control transgenic plants expressing the empty vector (Figure 6B, Additional file 1: Figure S4). Quantitative RT-PCR analysis showed that the expression of *OsPUB15* in randomly selected *OsPUB15*ox rice lines were up-regulated compared with that in control plants (Figure 6C). These results indicate that overexpression of *OsPUB15* leads to rice plants death as a result of uncontrolled propagation of spontaneous lesions.

To explore the biochemical mechanisms underlying the development of aggressive lesions in *OsPUB15*ox seedlings, we evaluated the expression of several histochemical markers in *OsPUB15*ox plants and control transgenic plants. When stained with trypan blue, an indicator of irreversible membrane damage or cell death, cells at the lesion sites of *OsPUB15*ox leaves exhibited deep blue in color whereas the cells in the leaves of control transgenic plants did not (Figure 6D). Moreover, the DAB (diamino benzidine) and NBT (nitro blue tetrazolium) staining analyses revealed that the accumulation of reactive oxygen species (ROS), such as H_2_O_2_ and O_2_^−^, were closely correlated with lesion formation in *OsPUB15*ox leaves. On the contrary, no ROS production was detected in leaves of the control transgenic plants (Figure 6D). These results reveal that the development of cell death in *OsPUB15*ox plants is likely resulted from excessive accumulation of ROS.

### Overexpression of *OsPUB15* activates rice defense responses constitutively

In most cases, the presence of lesion mimic cell death in plant is correlated with elevated expression of pathogen-related (PR) genes and enhanced resistance to pathogens [9,50]. Thus, we measured the transcript levels of four *PR* genes, *PR1a*, *PR1b*, *PR10* and *PBZ1*, by reverse transcription polymerase chain reaction (RT-PCR) analysis. We found that the expression of all these genes were up regulated in the *OsPUB15*ox plants both before and after lesion appearance (Figure 6E).

To test whether over-expression of *OsPUB15* enhances plant resistance to blast disease, we inoculated the *OsPUB15*ox plants and the control plants with seven *M. oryzae* isolates, Zhong-10-8-14, ZB13, ZB15, CH706, 99-26-1, 99-26-2 and 97-27-2, respectively. We found that, compared to the control transgenic plants, the *OsPUB15*ox plants confer enhanced resistance to all of the tested blast isolates (Figure 6F). Taken together, these findings suggest that overexpression of *OsPUB15* constitutively activates the basal defense responses against diverse isolates of *M. Oryzae* in rice.

## Discussion

Rice blast, caused by the most devastating rice pathogen, is a severe threat to global rice production [43]. More than 20 rice blast resistance genes have been cloned in the past years. However, only a few rice blast resistance genes, such as *Pita*, *Pb1* and *Pit*, have been well studied with the immediate downstream components identified [51-53]. In this study, we characterized the rice U-box/ARM repeat protein OsPUB15, an interacting component of rice blast resistance protein PID2. We found that the kinase domain of PID2 was able to interact directly with and transphosphorylate OsPUB15. We also found that the phosphorylation by PID2K was required for OsPUB15 to function as an active E3 ligase under our experimental conditions. Moreover, the fact that the *OsPUB15*ox plants exhibited spontaneous cell death phenotype and conferred enhanced basal defense suggest that OsPUB15 regulates plant PCD and innate immunity.

### OsPUB15 is a key factor involved in plant cell death and defense responses

Plant U-box/ARM repeat proteins have been studied extensively in connection with plant defense responses and cell death. SPL11 was the first characterized PUB E3 ligase involved in cell death and defense in rice. The *spl11* mutant developed spontaneous cell death in leaves and conferred enhanced resistance to multiple fungal and bacterial pathogens, suggesting that SPL11 might serve as a negative regulator of plant PCD and pathogenic defense [42,54]. As the closest *Arabidopsis* ortholog of rice SPL11, the AtPUB13 was also found to be involved in the regulation of cell death and plant defense [36]. The U-box E3 ligases CMPG1 and ACRE276 were characterized as positive regulators of hypersensitive response in tobacco [40,41]. In addition, the *Arabidopsis PUB17*-knockout mutants conferred compromised resistance to avirulent *Pseudomonas syringae pv. Tomato* [40]. On the contrary, the *Arabidopsis* triple mutant *pub22*/*pub23*/*pub24* was reported to display enhanced resistance to diverse pathogens, accompanied with oxidative burst and plant cell death [39]. Moreover, it is reported that the homozygous *pub44*/*pub44* mutant exhibited a seedling lethal phenotype resulting from widespread cell death lesions [38].

We investigated the biological function of *OsPUB15* using transgenic approach. The plants over-expressing *OsPUB15* exhibited cell death phenotype and conferred enhanced resistance to many *M. oryzae* isolates (Figure 6B, F). Interestingly, the excessive accumulation of ROS was observed in the lesion leaves of the *OsPUB15*ox plants (Figure 6D). It is likely that the high level of ROS in transgenic plants induces the abnormal phenotype and increases the resistance to pathogens, as rapid generation of ROS is a well-known process involved in plant PCD and innate immunity [55-57]. However, we failed to obtain transgenic rice lines with significantly decreased expression of *OsPUB15* by trying to introduce two *OsPUB15*-RNAi constructs (targeting different parts of the *OsPUB15* mRNA) individually into Pid2 rice calli or TP309 calli, respectively. It is likely that the potential regenerated plant or positive rice calli with drastically reduced expression of *OsPUB15* (attribute to the strong driving effect of the rice actin promoter for *OsPUB15*-*RNAi*) could not survive. This explanation is also supported by the findings that the homozygous T-DNA insertion mutant of *OsPUB15* in the rice variety Dongjin exhibits a seedling lethal phenotype [32]. Interestingly, the ROS accumulation and its impact on plant cell death were also found in the *OsPUB15*-knockout mutants [32]. Collectively, these results indicate that both up- and down- regulated expression of *OsPUB15* is able to induce accumulation of ROS and ultimately leading rice plants to death at seedling stage. Thus, we believe that the steady-state expression of *OsPUB15* is essential for plant survival and normal development. Additionally, *OsPUB15* is also involved in the responses against abiotic stresses as the expression of *OsPUB15* was up-regulated upon salt or drought stresses (Additional file 1: Figure S5). Taken together, our study reveals that OsPUB15 works as a key regulator for various ROS-related signaling pathways, including plant innate immunity, PCD and abiotic stresses.

### OsPUB15 is a substrate of the receptor-like kinase PID2

The rice U-box/ARM repeat protein OsPUB15 was previously isolated as one of the PID2K-interacting proteins through a yeast two-hybrid screening strategy [45]. By using the GST pull-down and BiFC approaches, we confirmed the interaction between OsPUB15 and PID2K *in vitro* and *in vivo* (Figure 2B, D). Furthermore, we found that the ARM repeat domain of OsPUB15 is essential for such interaction (Figure 2B, D), which is well consistent with the defined protein-protein interaction function of this domain [23,24].

The previous study has shown that PID2 is a transmembrane receptor-like kinase (RLK) containing a putative intracellular serine/threonine kinase domain [44]. Many RLKs with such cytosolic kinase domains, such as XA21 [58], OsSERK2 [59], BRI1 [60], BAK1 [61], EFR [62] and FLS2 [63], have been reported to possess kinase activity. Similarly, our present work reveals that PID2K is an active kinase (Figure 3). Moreover, we also found that PID2K is able to transphosphorylate OsPUB15 *in vitro* (Figure 3). The results that PID2K interacts directly with and transphosphorylates OsPUB15 support the notion that OsPUB15 is a substrate of PID2.

### E3 ligase activity of OsPUB15 relies on its phosphorylation

As is known, whether the E3 ligase activity of a protein could be successfully detected *in vitro* relies on the specific E2 enzyme(s) used for the ubiquitination assay. For example, the polyubiquitination of the E3 ligase OsPUB73 could be detected in the presence of the *Arabidopsis* E2 enzyme AtUBC8 or AtUBC9 rather than AtUBC7 [23]. Moreover, the U-box/RING proteins h-Goliath and AvrPtoB were able to exhibit strong ubiquitination activity when respectively incubated with the human E2 UBCh5c whereas they failed to display detectable ubiquitination activity when incubated with UBCh5b [64,65]. In addition, Park *et al*. found that out of the four E2s used (AtUBC5, AtUBC8, AtUBC10 and UBCh5c), only UBCh5c (also named hUBC5c) is effective in testing the E3 ligase activity of OsPUB15 [32]. Unfortunately, UBCh5c is not available for the OsPUB15 ubiquitination assay in our study. It seems that such E2 preference in ubiquitination assay may explain why we failed to detect the E3 ligase activity of bacterially expressed OsPUB15 even though a total of 11 E2s were used. Interestingly, after pre-incubating the bacterially expressed OsPUB15 with total protein extracts of rice or pre-phosphorylated by PID2K (Figure 4), we successfully detected the E3 ligase activity of OsPUB15. Our results suggest that the E3 ligase activity of OsPUB15 depends on its phosphorylation under our experimental conditions. In addition, we believe that the pre-incubation strategy used in this study would be adaptable to other prokaryotically expressed plant proteins for their *in*-*vitro* ubiquitination assays, especially when the most suitable E2s are not available or the post-translational modification is required.

### The mechanism for OsPUB15 to regulate plant cell death and defense responses

In this study, we extensively characterized the interaction between PID2K and OsPUB15 (Figure 2). We also found that PID2K is able to form homo-dimers in rice protoplast cells (Additional file 1: Figure S6). However, unlike PID2K, which lacks of the transmembrane and extra-cellular domain, the full-length PID2 (FL-PID2) is not able to form homo-dimers or interact with either OsPUB15 or OsPUB15C (Additional file 1: Figure S7). This indicates that the binding ability of PID2K with its partners is abolished in the native status of FL-PID2. We deduced that the binding sites of the native protein FL-PID2 in its kinase domain might not be well exposed and binding with a specific ligand (for example, the elicitors from blast pathogens) would be required for its conformational change enabling PID2 to homodimerize and/or to interact with its substrate(s), such as OsPUB15.

**Figure 7:**
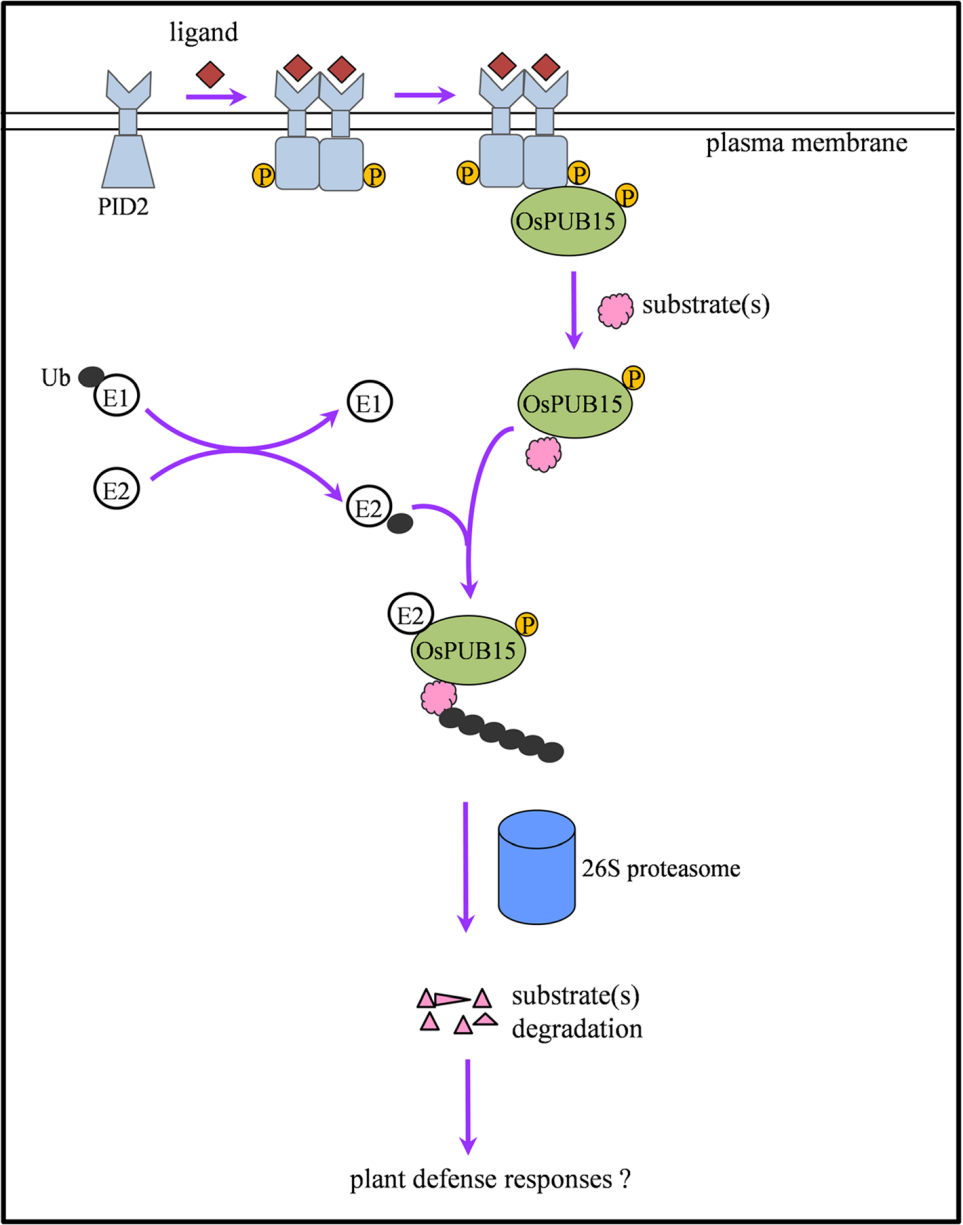
**A proposed model for OsPUB15 to regulate PID2**-**mediated signaling.** The unknown ligand from pathogen is likely able to activate the immune receptor PID2 by changing its conformation, which leads directly to the homodimerization and autophosphorylation of PID2. Such phosphorylated form of PID2 then recruits and transphosphorylates OsPUB15. The phosphorylated OsPUB15 serves as an active E3 ligase to mediate the degradation of unknown substrate(s), which is/are likely involved in PID2-mediated immune responses.

Many previous studies have revealed that homo-dimerization is required for some RLKs to be auto-phosphorylated in order to activate their full kinase activity, and in turn, the downstream signaling pathways in animals and plants [66-71]. According to this notion, we deduced that the homodimerization and autophosphorylation of PID2 might be required for the activation of PID2-mediated immune signaling. Collectively, we propose a model to summarize these results (Figure 7). In the absence of a corresponding ligand, the immune receptor PID2 is in a stable inactive state. The perception of the ligand by its extracellular B-lectin domain activates PID2 and promotes its homodimerization and autophosphorylation, as well as the recruitment and phosphorylation of cytosolic OsPUB15. After that, the phosphorylated OsPUB15 release E3 ligase activity to mediate the ubiquitination and degradation of its substrate(s), which might be -involved in regulating rice immune responses. Unfortunately, we could not provide genetic evidences to support this model as we failed to obtain the Pid2 plants silenced for *OsPUB15*. In future study, it will be interesting to determine the biological functions of OsPUB15 in PID2-mediated immunity using alternative genetic approaches.

Previous studies indicated that PID2 belonged to the non-RD subclass of RLKs [45,72], which is characterized by carrying an uncharged residue (such as cysteine, glycine, phenylalanine or leucine) in place of the conserved arginine (R) located just before the catalytic aspartate (D) residue with known roles in regulation of innate immunity in both animals and plants [73]. Besides PID2, the well-characterized immune receptors, XA21 and FLS2, also belong to non-RD kinases [73-75]. Interestingly, both XA21 and FLS2 also interact directly with their respective plant E3 ligases to regulate plant defense responses [76,77]. Thus, we suggest that the signal pathway mediated by interactions between non-RD kinases and E3 ligases responsible for regulating innate immunity might broadly exist in plant kingdoms.

More interestingly, the transgenic plants over-expressing *OsPUB15* but lacking of functional *Pid2* constitutively exhibit enhanced disease resistance to blast pathogens, accompanied with typical immune responses, such as rapid production of ROS and up-regulated expression of *PR* genes (Figure 6). These findings suggest that *OsPUB15* also regulates plant basal defense responses in a *Pid2*-independent manner. Together with its involvement in plant cell death, salt and drought stresses, our study reveals that OsPUB15 functions in many biological processes in rice, including plant growth, biotic and abiotic stresses.

## Conclusions

In this study, we confirmed that OsPUB15, a U-box/ARM repeat E3 ligase, was a binding partner of rice blast resistance protein PID2. In addition, we found that the kinase domain of PID2 was an active kinase capable of phosphorylating OsPUB15 and OsPUB15 was a substrate of PID2. We also found that rice plants over-expressing *OsPUB15* displayed spontaneous cell death, excessive accumulation of ROS, increased expression of pathogenesis-related genes and enhanced resistance to different blast strains. Taken together, our study demonstrates that OsPUB15 interacts with PID2 and plays significant roles in plant cell death and defense responses.

## Methods

### Plant materials and growth conditions

The rice (*Oryza sativa* L.) subspecies *indicia* cultivar Digu (*Pid2*/*Pid2*) conferring resistance to *M. oryzae* isolate ZB15 [44] and a susceptible *Oryza sativa* L. subsp. *japonica* ‘Taipei309’ (TP309) carrying homozygous *pid2* were used in this study. For expression profiling analysis of *OsPUB15*, the rice plants were cultivated in the farm field of the Institute of Genetics and Developmental Biology, Chinese Academy of Sciences in Beijing. To examine their response to blast fungi or the abiotic stresses, the rice seedlings were grown in the greenhouse at 25-28°C under a 16 h light/8 h dark regimen.

### *In vitro* GST pull-down assays

All primers used in this study are listed in Additional file 1: Table S1. The nucleotide sequence of PID2K was amplified from *Pid2*-cDNA [44] using primers #1 and #2, and the PCR product was cloned into the shuttle vector pEGH in frame with GST to generate GST-PID2K construct. OsPUB15 (primers #3 and #4), OsPUB15N (primers #3 and #5) and OsPUB15C (primers #6 and #4) fragments were separately amplified from FL (full length) -cDNA of *OsPUB15* and cloned into pET-30a (Novagen) in frame with 6 × His tag to generate His-OsPUB15, His-OsPUB15N and His-OsPUB15C constructs, respectively. Afterwards, these constructs were transformed into host strains, respectively. GST-PID2K was induced to largely express (2% D-Galactose, 14h, 30°C) in yeast strain Y258 and purified by affinity chromatography using Glutathione Sepharose 4B (GE Healthcare). The His-tagged proteins were individually induced to express (0.1 mM isopropyl β-D-thiogalactosiadse, overnight, 28°C) in *E. coli* strain Transetta (DE3) (TransGen Biotech, China) and purified with MagneHis™ Ni-Particles (Promega). The eluted His-tagged proteins were incubated with purified GST-PID2K or GST alone bound to glutathione beads, respectively. After 4 h of incubation at 4°C, the beads were extensively washed four times with GST binding buffer (PBS, pH 7.3). Components bound to the beads were eluted by boiling in SDS sample buffer, and then separated on a SDS-PAGE gel and immunoblotted with anti-OsPUB15 (Beijing Protein Innovation Co., Ltd., China) or anti-GST antibody (Beijing Protein Innovation Co., Ltd., China).

### *In vitro* phosphorylation assays

Two additional His-tagged OsPUB15 variants, His-OsPUB15C-1 (primers #7 and #4) and His-OsPUB15C-2 (primers #8 and #4) were generated and used in this analysis. The generation, expression and purification of both proteins were performed using similar strategies as those of His-OsPUB15. The autophosphorylation assay of purified GST-PID2K and transphosphorylation assays of the candidate substrates by GST-PID2K were performed according to the procedure as described previously [58].

### E3 ubiquitin ligase activity assays

The bacterially expressed His-OsPUB15, the pre-incubated His-OsPUB15 and the phosphorylated His-OsPUB15 were used in the assays. The pre-incubated His-OsPUB15 was obtained by incubating the bacterially expressed His-OsPUB15 bound to the MagneHis™ Ni-Particles with the total rice protein extracts [40 mM Tris–HCl, pH 7.4, 5 mM MgCl_2_, 5 mM ATP, 1 mM PMSF, 1X Protease Cocktail (Roche)] for 1 h at room temperature followed by extensively washing in PBS buffer. The bacterially expressed His-OsPUB15 and its phosphorylated form used in these assays were obtained as described above. The crude extract containing recombinant wheat E1 (GI: 136632), human E2 (UBCh5b), and purified *Arabidopsis* ubiquitin fused with the His tag were also used in these assays. The rice E3 ligase SDIR1 [78] was used as a positive control. The assays were conducted as described previously [79]. After the reactions, the resulting proteins were subjected to protein blot analysis using anti-Ub (produced by the laboratory of Prof. Qi Xie, Institute of Genetics and Developmental Biology, Chinese Academy of sciences) or anti-OsPUB15 antibody.

### Subcellular localization and BiFC assays in rice protoplasts

Rice protoplasts were prepared as described previously [80]. The coding sequences of OsPUB15 (primers #3 and #9) and PID2K (primers #10 and #11) without the stop codons were amplified and subcloned into the pBI221-GFP vector under the control of the cauliflower mosaic virus (CaMV) 35S promoter and in frame with the green fluorescent protein (GFP) to create 35S::OsPUB15-GFP and 35S::PID2K-GFP constructs, respectively. These constructs and the control 35S::GFP vector were respectively co-transformed into rice protoplasts along with pSAT6-mCherry-VirD2NLS plasmid [81] by polyethylene glycol-mediated transformation [80]. After incubation at 28°C in the dark for 14 h, the florescence signals were monitored with a confocal laser scanning microscope (LSM 510 META; Zeiss, Goettingen, Germany).

The BiFC vectors were obtained from The *Arabidopsis* Biological Resource Center (http://abrc.osu.edu/). For BiFC assays, the coding regions of PID2 (primers #12 and #13), PID2K (primers #14 and #15), OsPUB15 (primers #16 and #17), OsPUB15N-1 (primers #16 and #18) and OsPUB15C (primers #19 and #17) were separately subcloned into the BiFC vectors, including pSAT1-cEYFP-N1 and pSAT1-nEYFP-N1 [82], to generate PID2-nEYFP, PID2-cEYFP, PID2K-nEYFP, PID2K-cEYFP, OsPUB15-cEYFP, OsPUB15N-1-cEYFP and OsPUB15C-cEYFP constructs, respectively. Then the recombinant constructs in pairs were co-transformed into rice protoplasts together with pSAT6-mCherry-VirD2NLS plasmid and the signals were examined as described above.

### Generation of *OsPUB15ox* transgenic plants

The coding region of *OsPUB15* was amplified using primers #3 and #20 and cloned into the binary vector pTCK303 [83] to create the *OsPUB15* overexpression construct driven by maize *Ubiquitin* promoter. The construct was then introduced into embryogenic calli derived from TP309 seeds by Agrobacterium-mediated transformation method [84]. As a control, the empty pTCK303 vector was introduced into TP309 with similar procedures.

### Fungal treatments

Seven *M. oryzae* isolates, Zhong-10-8-14, ZB13, ZB15, CH706, 99-26-1, 99-26-2 and 97-27-2, were used in this study. The fungal spores of the *M. oryzae* isolates were harvested by washing with 0.25% Gelatin solution and diluted into the suspension at a concentration of 2 × 10^5^ spores/ml. To determine the transcript expression of *OsPUB15* in rice plants post inoculated with the *M. oryzae* isolate ZB15, such spore suspension were sprayed on four-leaf stage rice seedlings of Digu and TP309, respectively. On the other hand, to assess the response of *OsPUB15*ox seedlings to seven blast strains, 2 μl spore suspension were dropped onto the leaves of the two-week-old regenerated *OsPUB15*ox rice plants and the control plants grown on half-strength MS medium. Subsequently, the treated seedlings were put into a chamber kept at 28°C and 100% relative humidity in the darkness for 24 h and then transferred to a greenhouse with 16 h/8 h (day/night) photoperiod at 25-28°C in conditions of relatively high humidity as well. For the expression analysis of *OsPUB15*, the treated seedlings were sampled at 0, 1, 2, 3, 4, 5 and 6 days post inoculation (DPI); for the disease resistance analysis, the lesions on inoculated leaves of *OsPUB15*ox rice plants and the control plants were evaluated at 2 DPI, respectively.

### Gene expression analysis

Total RNA was extracted from plant tissues using Trizol reagent (Invitrogen, California, USA) according to the manufacturer’s manual. The Northern blotting analysis was conducted as described previously [44]. For RT-PCR analysis, the extracted RNA was treated with DNaseI (promega) to eliminate genomic DNA contamination and first-strand cDNA was synthesized from 2 μg of DNA-free RNA using reverse transcriptase (Promega, Madison, WI, USA) in a 25 μl reaction volume. Then 0.5 μl of each reverse transcription product was used as template for PCR reactions with gene-specific primers (primers #21 - #32 in Additional file 1: Table S1). The PCR reaction was conducted under the following conditions: 5 min at 94°C, 25 cycles (for the loading control *ACTIN1*) or 30 cycles (for the other genes) of 30 s at 94°C, 30 s at 60°C and 30 s at 72°C, followed by a 5 min extension at 72°C. The quantitative real-time PCR (qRT-PCR) analysis of *OsPUB15* expression with primers #21 and #22 was carried out as described previously [85]. The rice *ACTIN1* gene (primers #31 and #32) was used as an internal control for normalization of RNA samples.

### Histochemical analysis

Leaf samples of *OsPUB15*ox were collected for histochemical analysis after lesions appeared (about two weeks after the regenerated plantlets arisen). Leaves of the control plants at the same stage were used for comparison analysis. Trypan blue staining, DAB (diamino benzidine) staining and NBT (nitro blue tetrazolium) staining are indicative of cell death, H_2_O_2_ and O_2_^−^ accumulation, respectively. These staining analyses were performed following the protocols described previously [86].

### Abiotic stress treatments

Two-week-old TP309 rice seedlings grown in dishes were treated with 20% PEG (for drought stress) or 200mM NaCl solution (for salt stress). The shoot of seedlings collected at time-points 0, 0.5, 1, 2, 4, 8 and 24 h after treatment were used for RNA extracting for *OsPUB15* expression analysis.

### Phylogenetic analysis

Phylogenetic analysis was conducted using MEGA version 5 [87]. In brief, Full-length amino acid sequences of all PUB proteins were aligned by ClustalW using default parameters, and the phylogenetic tree was generated using a neighbor-joining algorithm with 1000 bootstrap replicates.

## Additional file

Additional file 1: Figure S1. Phylogenetic analysis of OsPUB15-related U-box/ARM repeat proteins in plants. **Figure S2.** OsPUB15 expressed in *E.coli* showed no detectable E3 ligase activity. **Figure S3.** Disease reactions of TP309 and Digu rice plants to *M.oryzae* isolate ZB15. **Figure S4.** Phenotype of transgenic rice plants over-expressing *OsPUB15*. **Figure S5.** The transcript expression of *OsPUB15* was induced by drought and high-salt stresses, respectively. **Figure S6.** The kinase domain of PID2 is able to form homodimers. **Figure S7.** Full length PID2 (FL-PID2) does not form homodimers or interact with OsPUB15 variants in rice protoplasts. **Table S1.** Primers used in this study.
