# Supplementary material for: The E3 ligase OsPUB15 interacts with the receptor-like kinase PID2 and regulates plant cell death and innate immunity
Source: BMC Plant Biol. 2015 Feb 13;15:49. doi: 10.1186/s12870-015-0442-4 (PMC4330927; doi:10.1186/s12870-015-0442-4)
Supplement: Additional file 1: Figure S1. — Phylogenetic analysis of OsPUB15-related U-box/ARM repeat proteins in plants. Figure S2. OsPUB15 expressed in E.coli showed no detectable E3 ligase activity. Figure S3. Disease reactions of TP309 and Digu rice plants to M.oryzae isolate ZB15. Figure S4. Phenotype of transgenic rice plants over-expressing OsPUB15. Figure S5. The transcript expression of OsPUB15 was induced by drought and high-salt stresses, respectively. Figure S6. The kinase domain of PID2 is able to form homodimers. Figure S7. Full length PID2 (FL-PID2) does not form homodimers or interact with OsPUB15 variants in rice protoplasts. Table S1. Primers used in this study. [file 12870_2015_442_MOESM1_ESM.doc]

**Additional file**

**Legends for additional file**

**Figure S1. Phylogenetic analysis of OsPUB15-related U-box/ARM repeat proteins in plants.** Full-length amino acid sequences of OsPUB15 and other PUB proteins with high similarities with the sequence of OsPUB15 from rice (Os), *Arabidposis* (At), tobacco (Nt), Brassica (Bn), potato (St), tomato (Sl) and pepper (Ca) were analyzed using MEGA 5 . The phylogenetic tree was generated using a bootstrap neighbor-joining tree applying 1,000 replicates. The solid diamond marked the position of OsPUB15.

**Figure S2. OsPUB15 expressed in *E.coli* showed no detectable E3 ligase activity.** His-OsPUB15 expressed and purified from *E.coli* was assayed for E3 activity in the presence of wheat E1, human E2 (UBCh5b) and His-Ub or in the absence of E1, E2 or His-OsPUB15. As a positive control, the E3 ligase SDIR1 was included in the assay. After the reactions, samples were resolved by 12% SDS-PAGE and immunoblotted with anti-Ub (top panel) or anti-OsPUB15 (bottom panel) antibody.

**Figure S3. Disease reactions of TP309 and Digu rice plants to *M.oryzae* isolate ZB15.** The rice seedlings of TP309 and Digu were inoculated with *M.oryzae* isolate ZB15 and their symptoms were evaluated 6 days after inoculation.

**Figure S4. Phenotype of transgenic rice plants over-expressing *OsPUB15*.** (A) Two-week-old *OsPUB15*ox plants under sterile conditions develop cell death lesions spontaneously. (B) 30-day-old *OsPUB15*ox plants under sterile conditions display a seedling lethal phenotype. The plants expressing the empty vectors at the same stages were used as controls. Bars = 2 cm.

**Figure S5. The transcript expression of *OsPUB15* was induced by drought and high-salt stresses, respectively.** qRT-PCR analysis of *OsPUB15* expression levels in two-week-old TP309 seedlings at 0, 0.5, 1, 2, 4, 8 and 24 h after treatment with 20% PEG [for drought stress, (A) ] or 200 mM NaCl solution [for salt stress, (B)]. The expression level of *OsPUB15* at 0 h was set as 1.0. The expression level of rice *ACTIN1* gene was used as an internal control for normalization of the data. Data represent means ± SDs of three replicates.

**Figure S6. The kinase domain of PID2 is able to form homodimers.** The PID2K-nEYFP and PID2K-cEYFP constructs were co-transformed into rice protoplasts together with the nuclear marker mCherry-NLS. And then fluorescence signals were monitored by confocal microscopy. The panel from left to right shows confocal images of the EYFP signal (green), the mCherry signal (red), bright-field images and the overlaid images, respectively. Bars = 5 μm. The experiment was repeated at least three times with similar results.

**Figure S7. Full length PID2 (FL-PID2) does not form homodimers or interact with OsPUB15 variants in rice protoplasts.** FL-PID2 and the OsPUB15 variants were respectively fused to the inactive N-terminal (nEYFP) or the C-terminal (cEYFP) of EYFP. The pairs, PID2-nEYFP/PID2-cEYFP (A), PID2-nEYFP/OsPUB15-cEYFP (B) and PID2-nEYFP/OsPUB15C-cEYFP (C) were respectively co-transformed into rice protoplasts along with the nuclear marker mCherry-NLS. As a positive interaction control, the pair of PID2K-nEYFP/OsPUB15-cEYFP was co-transformed with mCherry-NLS (D). The panel from left to right shows confocal images of the EYFP signal (green), the mCherry signal (red), bright-field images and the overlaid images, respectively. Bars = 5 μm. These experiments were repeated at least three times with similar results.

**Table S1.** **Primers used in this study.**

**Supplementary Figures**

**Figure S1**

**Figure S2**

**Figure S3**

**Figure S4**

**Figure S5**

**Figure S6**

**Figure S7**

**Table S1.** **Primers used in this study**

| No. | Primer name | Primer sequence (5’-3’) |
| --- | --- | --- |
| 1 | PID2K-F | GCATCACCATCACCATCACGGTGGTGGTGCTGGTTCATCGGAAGATGATG |
| 2 | PID2K-R | AGGCAGATCGTCAGTCAGTCACGATGAATCATCTGGGACCAGAGAGCCTCA |
| 3 | OsPUB15-F | GGGGATCCATGGAAAATTTCTCCCCGA |
| 4 | OsPUB15-R | GGGAGCTCTCTCCTTGCTGAATTCCCG |
| 5 | OsPUB15N-R | AAGAGCTCATATAAAGGTCAGCTTCCGGGG |
| 6 | OsPUB15C-F | GGGGATCCATGAAATCCTTGAAATTG |
| 7 | OsPUB15C-1-F | TTGGATCCGTTACAGATGGGCCTTCAG |
| 8 | OsPUB15C-2-F | GGGGATCCAATGATAATAATAAGATTGCC |
| 9 | OsPUB15-GFP-R | TTGAGCTCGGTCTCCTTGCTGAATTCCCG |
| 10 | PID2K-GFP-F | TTGGATCCGCTGGTTCATCGGAAGATGATG |
| 11 | PID2K-GFP-R | TTGTCGACTCTGGGACCAGAGAGCCT |
| 12 | PID2-c/nEYFP-F | CCGAATTCATGCAAATGTGTGGATGGTT |
| 13 | PID2-c/nEYFP-R | AACCCGGGTCTGGGACCAGAGAGCCT |
| 14 | PID2K-c/nEYFP-F | TCGAATTCGCTGGTTCATCGGAAGATG |
| 15 | PID2K-c/nEYFP-R | AACCCGGGTCTGGGACCAGAGAGCCT |
| 16 | OsPUB15-cEYFP-F | CTGAGCTCAATGGAAAATTTCTCCCCGA |
| 17 | OsPUB15-cEYFP-R | TTCCCGGGTCTCCTTGCTGAATTCCCG |
| 18 | OsPUB15N-1-cEYFP-R | TTCCCGGGACTACGAACATCAGCCAATC |
| 19 | OsPUB15C-cEYFP-F | TTGAGCTCAATGAAATCCTTGAAATTG |
| 20 | *OsPUB15*ox-R | TCGAGCTCTCATCTCCTTGCTGAATTCCCG |
| 21 | *OsPUB15*RT-F | CTGTCGCTGCTAAGGGTAAT |
| 22 | *OsPUB15*RT-R | CCATTCACCGAAGAACTACCC |
| 23 | *PR1a*-F | CGTCTTCATCACCTGCAACTACTC |
| 24 | *PR1a*-R | CATGCATAAACACGTAGCATAGCA |
| 25 | *PR1b*-F | GGCAACTTCGTCGGACAGA |
| 26 | *PR1b*-R | CCGTGGACCTGTTTACATTTTCA |
| 27 | *PR10*-F | CCCTGCCGAATACGCCTAA |
| 28 | *PR10*-R | CTCAAACGCCACGAGAATTTG |
| 29 | *PBZ1*-F | GGCACCATCTACACCATGAAGCT |
| 30 | *PBZ1*-R | TTGGACATTTCTGCGGCTCTCA |
| 31 | *ACTIN1*-F | AGCAACTGGGATGATATGGA |
| 32 | *ACTIN1*-R | CAGGGCGATGTAGGAAAGC |

**References**

1. Tamura K, Peterson D, Peterson N, Stecher G, Nei M, Kumar S: **MEGA5: molecular evolutionary genetics analysis using maximum likelihood, evolutionary distance, and maximum parsimony methods**. *Mol Biol Evol* 2011, **28**(10):2731-2739.

2. Zhang Y, Yang C, Li Y, Zheng N, Chen H, Zhao Q, Gao T, Guo H, Xie Q: **SDIR1 is a RING finger E3 ligase that positively regulates stress-responsive abscisic acid signaling in Arabidopsis**. *Plant Cell* 2007, **19**(6):1912-1929.
